# Correlation between cystatin C-based formulas, Schwartz formula and urinary creatinine clearance for glomerular filtration rate estimation in children with kidney disease

**DOI:** 10.15171/jrip.2016.33

**Published:** 2016-06-19

**Authors:** Afshin Safaei-Asl, Mercede Enshaei, Abtin Heydarzadeh, Shohreh Maleknejad

**Affiliations:** ^1^Department of Pediatrics, Guilan University of Medical Sciences, Guilan, Iran; ^2^Department of Community Medicine, Guilan University of Medical Sciences, Guilan, Iran

**Keywords:** Child, Creatinine, Cystatin C, Glomerular filtration rate, Kidney disease

## Abstract

**Introduction:** Assessment of glomerular filtration rate (GFR) is an important tool for monitoring renal function.

**Objectives:** Regarding to limitations in available methods, we intended to calculate GFR by cystatin C (Cys C) based formulas and determine correlation rate of them with current methods.

**Patients and Methods:** We studied 72 children (38 boys and 34 girls) with renal disorders. The 24 hour urinary creatinine (Cr) clearance was the gold standard method. GFR was measured with Schwartz formula and Cys C-based formulas (Grubb, Hoek, Larsson and Simple). Then correlation rates of these formulas were determined.

**Results:** Using Pearson correlation coefficient, a significant positive correlation between all formulas and the standard method was seen (R^2^ for Schwartz, Hoek, Larsson, Grubb and Simple formula was 0.639, 0.722, 0.705, 0.712, 0.722, respectively) (*P*<0.001). Cys C-based formulas could predict the variance of standard method results with high power. These formulas had correlation with Schwarz formula by R^2^ 0.62-0.65 (intermediate correlation). Using linear regression and constant (y-intercept), it revealed that Larsson, Hoek and Grubb formulas can estimate GFR amounts with no statistical difference compared with standard method; but Schwartz and Simple formulas overestimate GFR.

**Conclusion:** This study shows that Cys C–based formulas have strong relationship with 24 hour urinary Cr clearance. Hence, they can determine GFR in children with kidney injury, easier and with enough accuracy. It helps the physician to diagnosis of renal disease in early stages and improves the prognosis.

**Implication for health policy/practice/research/medical education:**

Glomerular filtration rate (GFR) is a calculation that determines how well the blood is filtered by the kidneys, which is one way to measure remaining kidney function. Assessment of GFR is an important tool for monitoring renal function. GFR is best measured by injecting compounds such as inulin, chromium-EDTA or iohexol, however these techniques are complicated, costly, time-consuming and have potential side-effects. Cystatin C is cysteine proteases inhibitor and has a low molecular weight that freely filters across the glomerulus and is neither reabsorbed nor metabolized by the kidney. Regarding to limitations in available methods, in this study we intended to calculate GFR by cystatin C based formulas and determine correlation rate of them with current methods.

## Introduction

Glomerular filtration rate (GFR) is summation of filtration in all functional nephrons, thus it is used as the best estimation of kidney function (1,2). This amount depends on age, gender and body surface area (3).

An ideal marker for calculating GFR is a marker with constant production, water soluble, not binding to protein, not having tubular excretion or reabsorption, not having extra renal elimination or metabolism, accurate and reliable, and having rapid results, cost-effectiveness and availability (4,5).

GFR is best measured by injecting compounds such as inulin, radioisotopes such as 51Cr-EDTA or radiocontrast agents such as iohexol, however these techniques are complicated, costly, time-consuming and have potential side-effects (6,7).

In clinical use, GFR is measured by serum creatinine (Cr) or Cr clearance that requires accurate urine collection over a long time. It is impossible in non-toilet trained children (without using a urinary catheter) and is challenging in other children (8,9).

Schwartz formula which is used widely in pediatrics is calculated based on serum Cr too (10-13).

Cys C is cysteine proteases inhibitor and has a low molecular weight (approximately 13.3 kDa) as a chain of 120 amino acids. It is encoded by the CST3 gene (5,9).

It is produced by all nucleated cells freely filters across the glomerulus and is neither reabsorbed nor metabolized by the kidney (14). Cr measurement has some problems. It is very variable and depends on age, gender, diet and muscle mass (5). Indeed it is influenced by factors other than kidney function (15,16). Measurement of Cys C is available increasingly and leads to rapid and accurate results (5). Serum Cys C has a steady level after first year of life (2), but Cr level increases until puberty that makes interpretation problematic for pediatric patients (17-20). Although in the presence of some conditions such as diabetes with ketonuria, increases of C-reactive protein (CRP), glucocorticoid therapy, cancer and thyroid dysfunction , serum level of Cys C is not reliable (21-28).

## Objectives

In this study we used Hoek, Larsson, Grubb and Simple formulas. Our gold standard test is 24 hour Cr clearance. Schwartz formula which is used widely in pediatric population is calculated too. Then the correlation between Cys C-based formulas and other methods was assessed. Early diagnosis of decreased kidney function, by only one serum sampling for Cys C is valuable.

## Patients and Methods

We studied 72 patients with renal diseases (including reflux nephropathy, nephrotic syndrome, obstructive uropathy and hereditary renal diseases) that referred to 17 Shahrivar hospital, including 38 boys and 34 girls of 2-14 years old.

Volume and Cr of 24 hour urine sample were measured. Cr (in serum and urine) and Cys C level were measured respectively by modified Jaffe method and enzyme immunoassay (ELISA). We recorded patients’ information including age, gender, height and type of kidney disease. For excluding criteria we measured fasting blood sugar, ketone in urine (only in diabetic patients), CRP, T4 and TSH. Exclusion criteria consisted of diabetes patients with ketonuria, elevated CRP level, glucocorticoid therapy, known cancer, thyroid dysfunction (hypothyroidism or hyperthyroidism) (24-28). Twenty-four hour urinary Cr clearance was our gold standard test which determined by this formula: C_Cr_ = U_Cr_ V/P_Cr_ and reported by ml/min (29-32).

Schwartz formula: GFR = K ×Height (cm)/S_Cr_ (mg/dL)

GFR also determined by Cys C-based formulas:

Hoek formula: GFR = -4.32+ (80.35 × 1/cystatin C (mg/l)

Larsson formula: GFR = 77.24 ×cystatin C (mg/l)­^-1.2623^

Grubb formula: GFR = 89.12× cystatin C (mg/l)^-1.1675^

Simple formula: GFR = 100/ cystatin C (mg/l)

Then the correlation between each of these results and 24 hour urinary Cr clearance and Schwartz formula were estimated.

### Ethical issues

1) The research followed the tenets of the Declaration of Helsinki; 2) Informed consent was obtained; and 3) the research was approved by the Ethics Committee of Guilan Univer­sity of Medical Sciences.

### Statistical analysis

Data analysis was done by SPSS and R^2^ between the mentioned methods were calculated by Pearson correlation coefficient. Then linear regression test was conducted and y-intercept was calculated that revealed the presence of overestimation or underestimation of the formulas in comparison with gold standard test and *P* value < 0.05 was recognized statistically significant.

## Results

**Figure 1:**
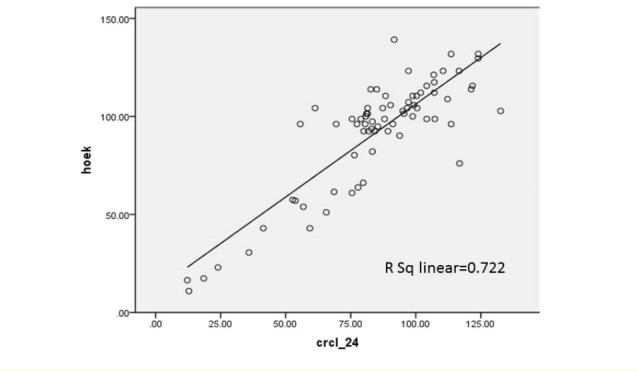
Correlation between GFR estimated from Hoek formula and 24 hour urinary Cr clearance.

**Figure 2:**
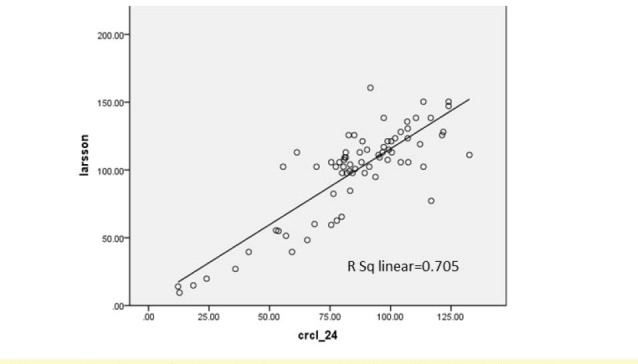
Correlation between GFR estimated from Larsson formula and 24 hour urinary Cr clearance

**Figure 3:**
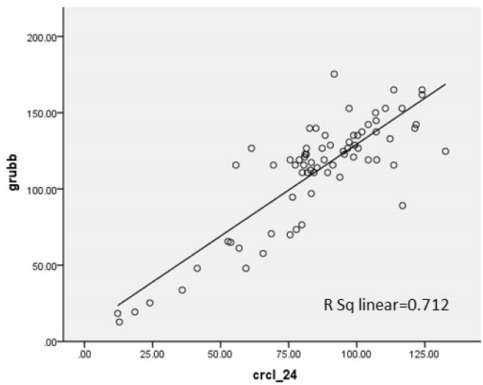
Correlation between GFR estimated from Grubb formula and 24 hour urinary Cr clearance.

**Figure 4:**
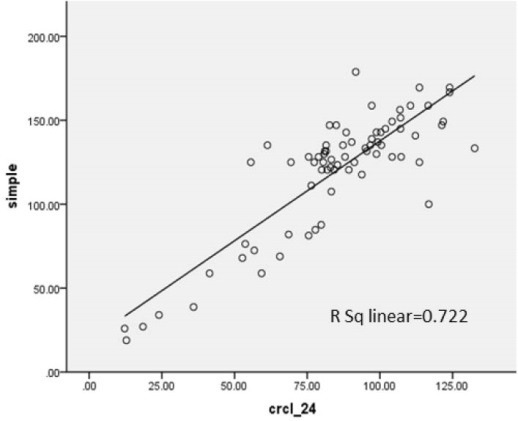
Correlation between GFR estimated from Simple formula and 24 hour urinary Cr clearance.

Seventy-two patients with renal disease were studied. Tirty-eight patients (52.8%) were boys and 34 patients (47.2%) were girls. Their age ranged from 2 to 14 years old (7.92 ± 3.79 years old).Their age ranged from 2 to 14 with the average of 7.92 ± 3.79 years old. The Underlying renal diseases were 14 nephrotic syndrome (19.4%), 12 congenital malformation including hypoplasia or agenesis (16.7%), 10 nephrolithiasis (13.8%), 9 reflux nephropathy (12.5%), 8 glomerulonephritis (11.1%), 6 anatomic disorders like ureteropelvic junction obstruction, ureterovesical junction obstruction, posterior urethral valve (8.3%), 5 urinary tract infection (UTI) (6.9%), 4 hereditary kidney disease including Autosomal recessive polycystic kidney disease (ARPKD) and medullary sponge kidney (MSK) (5.5%), 2 neurogenic bladder (2.8%) and 2 had Barter syndrome (2,8%). In this study, the value of Cys C level was 1.029 ± 0.79.24 mg/l. Urinary Cr clearance that was gold standard test for estimation of GFR in this study was 85.11 ± 25.72 ml/min/BSA (Tables 1 and 2). Using Pearson correlation coefficient, there was positive correlation in all methods. In correlation between 24 hour urinary Cr clearance and Schwartz formula, R^2^ = 0.639. It means that GFR calculated from Schwartz formula can predict about 64% of the variance of GFR calculated from Cr clearance test. Correlation rate between Hoek, Larsson, Grub, Simple GFRs and Schwartz formula was 0.651, 0.627, 0.636 and 0.655, respectively. Correlation rate between Hoek, Larsson, Grub, Simple GFRs and Cr clearance was shown in Figures 1-4.

**Table 1:** GFR calculated from all methods

**Method**	**Max**	**Min**	**Mean ± SD**
CrCl_24_	132.6	12.2	85.11 ± 25.79
Schwartz	14.03	153.12	96.55 ± 26.51
Hoek	139.16	10.93	92.2 ± 28.78
Larsson	139.16	10.93	98.98 ± 28.78
Grubb	175.37	12.8	111.51 ± 36.86
Simple	178.87	18.9	120.02 ± 36.12

GFR unit in all above methods is ml/min/1.73 m^2^.

**Table 2:** Y-intercept and corrected R between each method and gold standard test (Cr clearance)

**Method**	**Constant B±SE**	**P value**	**Corrected R**	**P value**
Schwartz Formula	26.62±6.56	<0.001	0.799	<0.001
Hoek Formula	11.52±6.25	0.07	0.85	<0.001
Larsson Formula	3.57±7.69	0.64	0.84	<0.001
Grubb Formula	8.88±8.15	0.28	0.84	<0.001
Simple Formula	18.74±7.84	<0.05	0.85	<0.001

## Discussion

In this study, correlation between Hoek, Larsson, Grub, Simple GFRs and Cr clearance was 0.712, 0.705, 0.722 and 0.722, respectively. R^2^, the prediction percentage of one variable from another, is interpreted this way; R^2^lower than 30%, 30%-49.99%, 50%-69.99% and more than 70% is indicative of lack of correlation, low, intermediate and high correlation between two variables respectively. This study shows a high correlation between Cys C based formulas and Cr clearance. On the other hand, there is intermediate correlation between these formulas and Schwartz formula. Also we used linear regression test and y-intercept. Y-intercept shows that we need to add this amount to the product of regression coefficient in GFR calculated from each method, then we can predict GFR from Cr clearance test. There is some difference between GFR calculated by Cys C formulas and Cr clearance, but after determining *P* value, it was demonstrated that these differences about Grubb, Hoek and Larsson formulas were statistically insignificant. In other words, these formulas can estimate GFR according to Cr clearance accurately.

Regarding Simple and Schwartz formulas, this difference was statistically significant and it means that these formulas overestimate GFR. In this study we used Cr clearance (in 24 hour urine) as gold standard test, but in most other studies, clearance of exogenous materials like 51Cr-EDTA (19,33,34) and 99m TC-DTPA (35), was used for gold standard test, which has more accuracy. In a similar study in Iran, Japan and France they used Cr clearance for gold standard test too (36-38) that shows less availability to these materials in our country and it leads to less diagnostic accuracy. In the study by Hoek et al in 2003, Hoek formula was introduced. This formula was compared with Cr-based formulas and showed more accurate results. However, the gold standard test was 125I-iothalamate, thus we cannot compare their result with our study (29). In the study of Larsson et al, in 2004, Larsson formula was introduced. The gold standard test was Iohexol clearance and correlation between Cyst C and standard test was determined (R^2^ = 0.91). Correlation between Cr and standard test (24 hour Cr clearance) was R^2^ =0.84 that was indicative of more correlation of Cys C. Also they distinguished that Larsson formula had more diagnostic value rather than isolated serum Cys C level (30). In 2005, Grubb formula was introduced. The gold standard test was Iohexol clearance, too. This formula had more diagnostic accuracy comparing to Cr-based formulas (31). In 2005 Perkins introduced Simple formula too. In that study the gold standard test was 125I-Iothalamate. There was high correlation between simple formula and standard test. (Spearman test; r = 0.77), however correlation between Cr-based formulas and standard test was low (Spearman test; r < 0.35) (32).

In study of Hojs et al (39) in 2009, gold standard test was 51 Cr-EDTA. Correlation between Larsson, Hoek, Grubb, Simple formulas and standard test was 0.895, 0.905, 0.899, and 0.906, respectively. All of the above formulas underestimated GFR, except Simple formula with little overestimation of GFR. Correlation in all formulas was high. They reported that Simple formula has acceptable diagnostic accuracy in clinical practice (39). In our study, as mentioned, a high correlation between Cys C-based formulas and our gold standard test (24 hour urinary Cr clearance) was seen.

## Conclusion

In conclusion using Cys C and mentioned formulas, which are more accurate than isolated serum Cys C level (40), we can determine GFR in children suspected to renal dysfunction easily, with high accuracy. Therefore, it is possible to early diagnosis and lead to better prognosis for patients.

## Limitations of the study

The study had some limitations such as small sample size in comparison of methods of calculat­ing GFR in them, thus we recommend conducting of similar studies as multicentric. Further studies with larger populations are suggested to better detect this aspect in children. One of the limitations of this study was the heterogeneity kidney disease among the study population.

## Acknowledgments

There is no doubt that conduction of the present study might not be feasible without cooperation of the patients, the respected colleagues. Authors wish to thank and appreciate efforts of Dr. Saba Hoda and Dr. Habib Habibzadeh for their assistance in performing the tests in Razi laboratory. In addition we are extremely grateful to the nurses of 17 Shahrivar children hospital for the support of the study.

## Authors’ contribution

ASA, AH and SM contributed to design and conducted the research. ME conducted data gathering and data interpretation. AS, AH and SM analyzed the data. All authors prepared the manuscript read, revised, and approved the final manuscript.

## Conflicts of interest

The authors report no conflicts of interest. The authors alone are responsible for the content and writing of the article.

## Ethical considerations

Ethical issues (including plagiarism, data fabrication, double publication) have been completely observed by the authors.

## Funding/Support

This study was financially supported by Guilan University of Medical Sciences (Grant # 650). This study extracted from residential thesis.

## References

[1] Pan CG, Avner ED. Glomerular filtration. In: Kliegman RM, Stanton BF, Geme III JW, Schor NF, ed. Nelson Textbook of Pediatrics. 19th ed. Philadelphia, PA: Elsevier Saunders; 2011.

[2] Hunley TE, Kan V, Ichikawa I. Glomerular circulation and function. In: Avner ED, Harmon WE, Niaudet P, Yoshikawa N, eds. Pediatric Nephrology. 6th ed. Berlin: Springer; 2009:31-64.

[3] Stevens LA, Levey AS. Measurement of kidney function. In: Singh AK, ed. Medical Clinics of North America. Philadelphia: WB Saunders; 2005:457. 10.1016/j.mcna.2004.11.009 15755462

[4] Assessment of kidney function. http://www.uptodate.com/contents/assessment-of-kidney-function. Accessed December 19, 2014.

[5] Bagshaw SM, Gibney RT (2008). Conventional markers of kidney function. Crit Care Med.

[6] Zahran A, El-Husseini A, Shoker A (2007). Can cystatin C replace creatinine to estimate glomerular filtration rate? A literature review. Am J Nephrol.

[7] Roos JF, Doust J, Tett SE, Kirkpatrick CM (2007). Diagnostic accuracy of cystatin C compared to serum creatinine for the estimation of renal dysfunction in adults and children--a meta-analysis. Clin Biochem.

[8] Viswanathan V, Snehalatha C, Nair MB, Ramachandran A (2005). Comparative assessment of cystatin c and creatinine for determining renal function. Indian J Nephrol.

[9] King AJ, Levey AS (1993). Dietary protein and renal function. J Am Soc Nephrol.

[10] Brion LP, Fleischman AR, McCarton C, Schwartz GJ (1986). A simple estimate of glomerular filtration rate in low birth weight infants during the first year of life: noninvasive assessment of body composition and growth. J Pediatr.

[11] Schwartz GJ, Feld LG, Langford DJ (1984). A simple estimate of glomerular filtration rate in full-term infants during the first year of life. J Pediatr.

[12] Schwartz GJ, Haycock GB, Edelmann CM, Spitzer A (1976). A simple estimate of glomerular filtration rate in children derived from body length and plasma creatinine. Pediatrics.

[13] Schwartz GJ, Gauthier B (1985). A simple estimate of glomerular filtration rate in adolescent boys. J Pediatr.

[14] Shemesh O, Golbetz H, Kriss JP, Myers BD (1985). Limitations of creatinine as a filtration marker in glomerulopathic patients. Kidney Int.

[15] Perrone RD, Madias NE, Levey AS (1992). Serum creatinine as an index of renal function: new insights into old concepts. Clin Chem.

[16] Branten AJ, Vervoort G, Wetzels JF (2005). Serum creatinine is a poor marker of GFR in nephrotic syndrome. Nephrol Dial Transplant.

[17] Bökenkamp A, Domanetzki M, Zinck R, Schumann G, Brodehl J (1998). Reference values for cystatin C serum concentrations in children. Pediatr Nephrol.

[18] Finney H, Newman DJ, Thakkar H, Fell JM, Price CP (2000). Reference ranges for plasma cystatin C and creatinine measurements in premature infants, neonates, and older children. Arch Dis Child.

[19] Helin I, Axenram M, Grubb A (1998). Serum cystatin C as a determinant of glomerular filtration rate in children. Clin Nephrol.

[20] Randers E, Krue S, Erlandsen EJ, Danielsen H, Hansen LG (1999). Reference interval for serum cystatin C in children. Clin Chem.

[21] Rule AD, Bergstralh EJ, Slezak JM, Bergert J, Larson TS (2006). Glomerular filtration rate estimated by cystatin C among different clinical presentations. Kidney Int.

[22] White C, Akbari A, Hussain N, Dinh L, Filler G, Lepage N, Knoll GA (2005). Estimating glomerular filtration rate in kidney transplantation: a comparison between serum creatinine and cystatin C-based methods. J Am Soc Nephrol.

[23] Pöge U, Gerhardt T, Stoffel-Wagner B, Klehr HU, Sauerbruch T, Woitas RP (2006). Calculation of glomerular filtration rate based on cystatin C in cirrhotic patients. Nephrol Dial Transplant.

[24] Holmquist P, Torffvit O, Sjöblad S (2003). Metabolic status in diabetes mellitus affects markers for glomerular filtration rate. Pediatr Nephrol.

[25] Knight EL, Verhave JC, Spiegelman D, Hillege HL, de Zeeuw D, Curhan GC (2004). Factors influencing serum cystatin C levels other than renal function and the impact on renal function measurement. Kidney Int.

[26] Risch L, Herklotz R, Blumberg A, Huber AR (2001). Effects of glucocorticoid immunosuppression on serum cystatin C concentrations in renal transplant patients. Clin Chem.

[27] Kos J, Stabuc B, Cimerman N, Brünner N (1998). Serum cystatin C, a new marker of glomerular: filtration rate, is increased during malignant progression. Clin Chem.

[28] Fricker M, Wiesli P, Brändle M, Schwegler B, Schmid C (2003). Impact of thyroid dysfunction on serum cystatin C. Kidney Int.

[29] Hoek FJ, Kemperman FA, Krediet RT (2003). A comparison between cystatin C, plasma creatinine and the Cockcroft and Gault formula for the estimation of glomerular filtration rate. Nephrol Dial Transplant.

[30] Larsson A, Malm J, Grubb A, Hansson LO (2004). Calculation of glomerular filtration rate expressed in ml/min from plasma cystatin C values in mg/l. Scand J Clin Lab Invest.

[31] Grubb A, Björk J, Lindström V, Sterner G, Bondesson P, Nyman U (2005). A cystatin C-based formula without anthropometric variables estimates glomerular filtration rate better than creatinine clearance using the Cockcroft-Gault formula. Scand J Clin Lab Invest.

[32] Perkins BA, Nelson RG, Ostrander BE, Blouch KL, Krolewski AS, Myers BD (2005). Detection of renal function decline in patients with diabetes and normal or elevated GFR by serial measurements of serum cystatin C concentration: results of a 4-year follow up study. J Am Soc Nephrol.

[33] Ylinen EA, Ala-Houhala M, Harmoinen AP, Knip M (1999). Cystatin C as a marker for glomerular filtration rate in pediatric patients. Pediatr Nephrol.

[34] Willems HL, Hilbrands LB, van de Calseyde JF, Monnens LA, Swinkels DW (2003). Is serum cystatin C the marker of choice to predict glomerular filtration rate in paediatric patients?. Ann Clin Biochem.

[35] Krieser D, Rosenberg AR, Kainer G, Naidoo D (2002). The relationship between serum creatinine, serum cystatin C and glomerular filtration rate in pediatric renal transplant recipients: a pilot study. Pediatr Transplant.

[36] Soleimani M, Zargar Shoushtari M, Shahrokh H, Habib Akhyari H, Kaffash Nayyeri R, Fereshtehnejad S (2009). Comparison study of the diagnostic values of serum cyctatin C and creatinine in the assessment of renal function in the early follow- up of renal transplant patients. Razi Journal of Medical Sciences.

[37] Uemura O, Ushijima K, Nagai T, Yamada T, Hayakawa H, Nabeta Y (2010). Reference serum cystatin C levels in Japanese children. Clin Exp Nephrol.

[38] Bacchetta J, Cochat P, Rognant N, Ranchin B, Hadj-Aissa A, Dubourg L (2010). Which creatinine and cystatin C equations can be reliably used in children?. Clin J Am Soc Nephrol.

[39] Hojs R, Bevc S, Ekart R, Gorenjak M, Puklavec L (2010). Serum cystatin C-based formulas for prediction of glomerular filtration rate in patients with chronic kidney disease. Nephron Clin Pract.

[40] Löfberg H, Grubb AO (1979). Quantitation of gamma-trace in human biological fluids: indications for production in the central nervous system. Scand J Clin Lab Invest.

